# The diagnostic and initial approach of the patient with non-alcoholic fatty liver disease: role of the primary care provider 

**Published:** 2019

**Authors:** Nicolás Salva-Pastor, Norberto C. Chávez-Tapia, Misael Uribe, Natalia Nuño-Lámbarri

**Affiliations:** 1 *Traslational Research Unit, Medica Sur Clinic & Foundation, Mexico City, Mexico*; 2 *School of Medicine, Benemérita Universidad Autónoma de Puebla, Puebla, Puebla, Mexico*; 3 *Obesity and Digestive Diseases Unit, Medica Sur Clinic & Foundation, Mexico City, Mexico*

**Keywords:** Fibrosis, Liver, Steatohepatitis, Steatosis

## Abstract

Non-alcoholic fatty liver disease (NAFLD) represents a broad spectrum of liver damage, ranging from simple steatosis to steatohepatitis and fibrosis; as well, there is a close association between NAFLD, obesity, metabolic syndrome and type 2 diabetes mellitus. There is a certain degree of uncertainty regarding the natural history and prognosis of NAFLD; however, several methods are currently used for its diagnostic approach. In the first instance, non-invasive tests could be used to identify patients at low risk of developing fibrosis and to establish more easily the need for a liver biopsy, whose accuracy in the evaluation of fibrosis has been questioned, mainly due to errors of intra and interobserver sampling, technical problems and cost, which limits its use. Therefore, it is essential to determine the diagnostic strategy for patients with NAFLD.

## Definition

According to the American Association for the Study of Hepatic Diseases, the American College of Gastroenterology and the American Association of Gastroenterology, evidence of hepatic steatosis is required to define non-alcoholic fatty liver disease (NAFLD), either by imaging or histology. It is also required that there are no other causes of steatosis or chronic liver disease, as well as the absence of significant alcohol consumption. The most common causes of hepatic steatosis are substantial alcohol intake, hepatitis C, steatogenic drugs, parenteral nutrition, Wilson's disease, and severe malnutrition. NAFLD includes a broad group of pathologies ranging from non-alcoholic fatty liver, non-alcoholic steatohepatitis (NASH), fibrosis, cirrhosis, and even hepatocellular carcinoma (HCC) (1,2).

NAFLD is defined as the presence of excessive hepatic fat content above 5%, without evidence of hepatocellular injury. NASH refers to the presence of hepatic steatosis and inflammation, additionally to parenchymal injury, with or without fibrosis.

NALFD is highly prevalent and results from excessive fat accumulation, mainly free fatty acids, triglycerides, and cholesterol. There is an increasing awareness interest in NAFLD because it is considered the leading cause of abnormal liver aminotransferase levels and chronic liver disease; moreover, the liver is targeted by signals from other tissues, including adipose tissue and the gut´s microbiota. Besides, it is considered an emerging health problem due to malnourishment or a high-fat diet intake, which is observed worldwide (1,3,4).

## Epidemiology

It is estimated that NAFLD has an overall prevalence of 25% (95% CI: 22.10-28.65), while NASH prevalence in patients with a previous histological diagnosis of NAFLD is 59%. On the other hand, the global prevalence of obesity in patients with NAFLD is 43%, whereas, in patients with the previous diagnosis of NASH, it is 81% (5). Besides, NAFLD is associated with an increased risk of morbidity and mortality due to cardiovascular diseases (1,6,7), being an independent risk factor that contributes to the progression of atherosclerosis (7).

**Risk factors**

There is a close association between NAFLD and obesity, metabolic syndrome, and type 2 diabetes mellitus, each with a prevalence of 43%, 51%, and 23% respectively. NAFLD global prevalence increased from 15% in 2005 to 25% in 2010, in parallel with obesity rates (8). It is also known that the composition of the diet, mainly the type of lipids and carbohydrates, has an essential role in the progression of NAFLD to NASH and fibrosis. Excess carbohydrate consumption has been extensively related to the development of NAFLD (9).

It has become clear that there are bidirectional links between NAFLD and type 2 diabetes mellitus, which share some aspects of its pathophysiology. Patients with NAFLD have a higher risk of developing diabetes, compared to patients without NAFLD (HR: 2.22, 95% CI: 1.84-2.60, I2= 79.2%) (10). For instance, the progression from NAFLD to NASH increases the prevalence of having diabetes to 43.63%. Insulin resistance and type 2 diabetes mellitus are among the most critical predictors of damage progression in advanced fibrosis and cirrhosis (2,5).

The presence of metabolic syndrome is a strong predictor of steatohepatitis and can be used to identify patients who persist with abnormal liver tests. However, a much more accurate diagnosis is obtained with a biopsy (8). Its prevalence in patients with NAFLD is 42.53% while in patients with NASH, it is 70.65% (5). Hyperlipidemia and hypertension are also significant risk factors for the development of cirrhosis due to NAFLD (11). The prevalence of hyperlipidemia/dyslipidemia in patients with NAFLD and NASH is 69.16% and 72.13% respectively, while the overall prevalence of hypertriglyceridemia in patients with NAFLD is 40.74% and in NASH patients it is 83.33%; in the other hand, the prevalence of hypertension in patients with NAFLD and NASH is 39.34 and 67.97% respectively (5).

There are four patient phenotypes according to the degree of obesity and the metabolic state: first the metabolically healthy and slender, second the metabolically healthy and obese, third the metabolically unhealthy and slim, finally the metabolically unhealthy and overweight. Among the four phenotypes, there are alterations in the pathways of inflammation; however, the pathophysiological mechanisms in NAFLD are not yet precise (12). The risk of developing NAFLD is higher in people classified as metabolically unhealthy, compared to those metabolically healthy. Using lean and metabolically healthy patients as a reference, an odds ratio is estimated for obese but metabolically healthy of 1.7 (CI 95%, 1.239 to 2.419), for the lean but metabolically unhealthy 1.8 (95% CI, 1.412 to 2.494) and for obese and metabolically unhealthy of 2.5 (95% CI, 1.699 to 3.681) (13).

**Natural history**

The advances in the understanding of this pathogenesis have revealed the complexity of the disease. The "two hits" hypothesis has been replaced by a "multiple impact" model that incorporates various processes, including lipotoxicity, activation of innate immunity and microbiota, in a context of environmental and genetic factors (6,14). There is a certain degree of uncertainty regarding the natural history and prognosis of NAFLD. The long evolution of NAFLD can, in a minority of patients, lead to steatohepatitis, which progresses more frequently to cirrhosis and hepatocellular carcinoma; however, progression to fibrosis in patients with NAFLD is rare (15). Is essential to note the association between NAFLD and NASH long-term prognosis, as well as the liver disease stage, since NASH is an increasingly common condition and one of the most important causes of chronic liver disease (9,16).

In long-term follow-up studies, only 1% of patients with NAFLD develop cirrhosis and die of causes related to the liver after an average of 15.6 years. On the other hand, 11% of patients with NASH develop cirrhosis, and 7.3% die of a liver-related cause, after a similar follow-up period. Leading to the concept that NAFLD is a relatively benign disease, whereas NASH represents the potentially progressive form of NAFLD to cirrhosis and its complications. The exact problem of Hepatocellular Carcinoma (HCC) related to NAFLD remains uncertain, but NAFLD will be the most common underlying etiologic risk factor for HCC; (9) in the United States is considered the third most common cause of HCC (1). Is estimated that the presence of NASH without cirrhosis carries a 2.5 times greater risk, (OR 2.61, 95% CI 1.27 to 5.35, P = 0.009, I2 = 95%) of developing HCC, in comparison with other chronic non-cirrhotic liver diseases. The prevalence of HCC in patients with NASH without cirrhosis is 38% (17).

The progression of NAFLD to fibrosis represents a complex interaction between genetic factors and extrinsic or intrinsic environmental agents. The estimated annual rate of evolution to fibrosis is 0.13 stages (CI 95%, 0.07-0.18), which corresponds to an average progression of one stage in 7.7 years (CI 95%, 5.5 -14.8 years); on the other hand, the rate of progress in NAFLD and NASH is estimated at 0.07 (95% CI, 0.02-0.11), and 0.14 (95% CI, 0.07-0.21) stages per year, which represents a progression of a stage in about 14.3 (95% CI, 9.1-50.0) and 7.1 (95% CI, 4.8-14.3) years, respectively. However, two distinct groups have been identified among the patients that develop hepatic fibrosis, the so-called fast progressors (progression from stage 0 to stages 3 or 4) and slow progressors (progress from stage 0 to stages 1 or 2), which is why the progress is not considered universal, nor linear (18).

The natural history of NAFLD is potentially modifiable through changes in the diet and the lifestyle; therefore, it is not necessarily a progressive condition.

## Evaluation of the patient with NAFLD

Several methods are used for the diagnostic approach of NAFLD. The fundamental problems are to differentiate NAFLD from NASH, and second, to stage the degree of hepatic fibrosis due to the fact patients with NASH/fibrosis have a higher risk of developing cirrhosis, liver failure and hepatocellular carcinoma (19). Liver biopsy is the gold standard for the diagnosis, differentiation and staging of steatosis and non-alcoholic steatohepatitis; (8,20–24) however, there is an great interest in non-invasive methods to identify advanced fibrosis in patients with non-alcoholic fatty liver (8).

Due to the high prevalence of NAFLD in the general population, non-invasive tests are first-line tools to evaluate patients and thus more easily establish the need for a liver biopsy (19).

**Laboratory studies**

There is an active search for cheaper and readily available laboratory tests for the detection of hepatic steatosis and fibrosis, as well as the estimation of severity. Multiple serological markers that reflect hepatic function have been used in combination to formulate diagnostic and prognostic scores as an alternative to liver biopsy (21,25).

**Platelet count**

Platelets have a well-known role in hemostasis, but they also participate in the liver inflammation process, which promote leukocyte recruitment through hepatic sinusoids, and activate effector cells. Some studies suggest that platelets play a role in hepatic fibrosis process by decreasing the expression of the transforming growth factor-beta (TGF-β) and increasing the expression of matrix metalloproteinases. Therefore, there is an inverse relationship between the progression of liver fibrosis and platelets. Patients with NAFLD have a low platelets count and increased plateletcrit, mean platelet volume, and the platelet distribution width. Indeed, steatohepatitis is associated with an elevation in the mean platelet volume, so the platelet count and all the platelet indexes are predictors of this disease. Their main advantage is that they are easy to measure routinely (25,26).

**Aminotransferases serum levels**

Within blood tests, alanine aminotransferase (ALT) or aspartate aminotransferase (AST) have been associated with inflammation and steatosis; however, each marker alone does not correlate with the degree of fibrosis (27). The reproducibility of measuring AST levels or platelet counts is questionable, (28) up to 80% of patients with NAFLD have AST and ALT levels within normal ranges, even in clinically significant advanced fibrosis due to NASH (29).

The AST/ALT index is considered an ideal biomarker for hepatic fibrosis, which increases with the progression of the disease. The APRI Index (AST to Platelet Ratio Index) has an accuracy of 0.85 for advanced fibrosis (stage 3-4) in patients with previous NAFLD (30). The AST/ALT relationship with the Fibrosis-4 (FIB-4) and NAFLD Fibrosis Score (NFS) can reliably exclude advanced fibrosis and reduce the need for liver biopsy in patients with NAFLD, regardless of the ALT levels. (29) The NFS, FIB- 4, and APRI, have an accuracy of 0.84, 0.85 and 0.80, respectively, and can be used for the non-invasive diagnosis of advanced fibrosis in the Mexican and Chilean population, mainly to discard advanced fibrosis with cut-off values: NFS> 0.676, FIB-4> 3.35, and APRI> 1 (31).

**FIB-4 INDEX**

The FIB-4 Index is calculated with four variables (age, AST, ALT, and platelet count) and it has high precision for advanced fibrosis in patients with NAFLD, also is a simple and inexpensive test, but the score is difficult to use for NASH diagnosis, and the diagnostic accuracy decreases with age (30). Recently, new cut-points have been proposed, which combine conventional and modified values, considering the age group to which they belong. In patients younger than 49 years, the cut-off point is 1.05 to 1.21, for 50-59 years is 1.24 to 1.96, for 60-69 years is 1.88 to 2.67, and those patients over 70 years the cut-off point is 1.95 to 2.67; these cut-off points improve the diagnostic accuracy of advanced fibrosis (32).

**NAFLD fibrosis score**

*NFS *is based on six variables (age, body mass index, hyperglycemia, platelet count, albumin, and AST/ALT ratio). NFS has an accuracy of 0.85 to predict advanced fibrosis. With a score of ≤-1.455, it has a sensitivity of 90% and a specificity of 60% to exclude advanced fibrosis, while a score ≥ 0.676 has a sensitivity of 67% and a specificity of 97% to identify the presence of advanced fibrosis (1,8). In patients with NAFLD, NFS is currently the most studied and validated biomarker. 

Among the different serum biomarkers studied in NAFLD, only NFS and FIB-4 have been validated externally more than once in diverse populations with consistent results. These tests work better to exclude advanced fibrosis-cirrhosis (with negative predictive values > 90%); therefore, could be used as a first-line classification to identify patients with low risk of having advanced fibrosis (28).

**Imaging techniques**

Imaging plays a vital role in the diagnosis of NAFLD, either in patients who were referred with abnormal liver function tests, by clinical suspicion (obesity, hyperlipidemia, type 2 diabetes mellitus) or when there are irregularities in the imaging studies performed for other reasons (33).

**Ultrasonography**

The ultrasonographic characteristics of hepatic steatosis are due to intracellular fat vacuoles, which generate an increase in the reflection of sound waves concerning the normal hepatic parenchyma, which results in higher echogenicity, visualized with a "bright liver" pattern (33,34).

The parameters used to evaluate liver steatosis by ultrasound are the difference between liver and kidney echogenicity, the deep penetration of the sound beam in the liver and the determination of blood vessels clarity. Frequently, fat deposition is diffuse; therefore, the liver will have a homogenous echogenic appearance; however, the right kidney that is located just below the right hepatic lobe can be used as a reference. In healthy patients, the liver will have an echogenicity similar to the renal cortex, contrary to a more marked contrast between the echogenicity of the liver and the adjacent renal cortex suggests hepatic steatosis. The increased reflection of sound waves due to the infiltration of fat in the liver can result in a thicker hepatic echotexture, a decrease in the penetration of the ultrasound beam of the deep part and a loss in the visualization of the right hemidiaphragm and the portal triad, which are evident by ultrasound in a healthy liver (33).

The severity of the disease is usually classified with a scale of four points: normal (grade 0), mild (grade 1), moderate (grade 2), and severe (grade 3). Mild steatosis is defined as a higher echogenicity of the hepatic parenchyma without darkening of the portal triad. Moderate steatosis is characterized by a higher echogenicity of the hepatic parenchyma that obscures the portal triad, whereas severe steatosis is considered when the liver is sufficiently echogenic to darken the diaphragm and limit the evaluation of the deep hepatic parenchyma by attenuating the ultrasound beam (16,33).

Ultrasound is easily accessible; however, when steatosis is less than 30%, the sensitivity is significantly reduced; therefore, it is not ideal for detecting the early stages of the disease (6,33). Because fibrosis may increase the echogenicity of the liver, the presence of the underlying chronic liver disease may reduce the accuracy of hepatic steatosis diagnosis. On the other hand, because the ultrasound has a qualitative nature and is a dependent operator, it is considered to have low sensitivity and specificity to diagnose and monitor NAFLD mild stages, it also does not allow to determine the presence of NASH or fibrosis (34–36).

**Transient elastography**

Transient elastography (TE, FibroScan®) is an ultrasound-based imaging technique that allows rapid measurements of liver stiffness, an indicator that is strongly related to the stage of liver fibrosis (35,36). The Controlled Attenuation Parameter (CAP) has been developed based on the properties of ultrasonic signals through the TE, which can be used in the detection and quantification of hepatic steatosis. Also, it offers the advantage that both steatosis and fibrosis can be assessed simultaneously, thus improving the ability of non-invasive methods in the detection and monitoring of NAFLD (35–37).

TE-based liver stiffness measurements using the M probe correlate with stages of fibrosis, particularly in severe fibrosis and cirrhosis. An essential limitation of TE is the high failure rate in patients with a BMI > 28 kg/m2 that limits the reliable measurement of liver stiffness and steatosis in a significant portion of obese patients with NAFLD, (38) nevertheless, with the development of the XL probe, (2.5 MHz transducer) a detection of hepatic steatosis and fibrosis can be detected in patients suffering from obesity, since it allows measurements between 35 and 75 mm in depth (28,36). It is essential to take into account that the stiffness values obtained with the XL probe are lower than those obtained with the M probe (with a median of 1.4 kPa) (28).

Before an ET, patients must have a fast of at least four hours. The procedure is performed in a supine position with the right arm wholly adducted, holding the breath for 10 seconds. The M probe (3.5 MHz) is applied to the area of the abdomen in the area of the right hepatic lobe; if necessary, when the device indicates it, patients are scanned again using the XL probe. A minimum of 10 measurements is taken to obtain the median hepatic stiffness in kilopascals (kPa) and the interquartile range (IQR) (38).

Through TE, significant hepatic steatosis is defined as a CAP value ≥238 dB/m, (36) while values between 10 and 15 kPa are suggestive of substantial fibrosis; however, additional tests are required for confirmation (39). 

The final result of a TE can be considered valid and reliable if the following criteria are met: At least ten valid measurements; a success rate (the ratio of valid measures to the total number of measures) greater than 60% and an interquartile/median range (IQR/M) less than 0.30 (28). 

It is convenient to determine the reliability according to the IQR/M, classifying as unreliable the evaluation with an IQR/M> 0.30, reliable if it is <0.30 and very reliable less than 0.10, the latter being the one with the highest precision. (36,40,41) Fibrosis diagnosis by TE has an accuracy of 0.67 (95% CI: 0.56-0.78), with a high negative predictive value for significant fibrosis (stages 2-3), severe fibrosis (stages 3-4) and cirrhosis, in addition to steatosis diagnosis using CAP (38).

The associated factors to consider evaluation as unsuccessful are: being female (OR 1.707, 95% CI 1.084-2.688, p= 0.02) and choosing the wrong size of the probe (OR 1.85, 95% CI 1.164-2.959, p= 0.009) (41). TE results may be difficult to obtain in obese patients, but also in patients with narrow intercostal space, however, in patients with ascites, it is almost impossible to obtain (28).

**Computed tomography**

The density of a structure reflected as brightness or darkness by computed tomography (CT) without contrast, is based on the radiation attenuation of the structure. On unenhanced CT, a healthy liver will have a slightly higher attenuation than the spleen so that it could serve as a reference. Therefore, it is inferred that the infiltration of fat into a soft tissue structure, such as the liver, decreases attenuation (33,34).

The decrease in hepatic attenuation concerning the spleen has a sensitivity of 88% to 95% and a specificity of 90% to 99% for fatty liver diagnosis, which is a criterion based on the detection of a liver density 10 Hounsfield units (HU) lower than splenic density. A hepatic attenuation below 40 HU on non-contrast CT is the most certain criterion to diagnose moderate to severe steatosis (33,35). Through volumetric tomography, the liver volume can be evaluated to predict the stage of fibrosis derived from NAFLD, in which the volume percentage of the caudate lobe has a high diagnostic yield (value of Az = 0.955) (22). The low accuracy of CT to detect a mild degree of hepatic steatosis suggests that this method may not be the most adequate to evaluate NAFLD (34).

**Magnetic resonance**

Methods based on magnetic resonance (MR) are increasingly used for the diagnosis of NAFLD. The use of an MR technique, either MR imaging (MRI), MR spectroscopy (MRS) or MR elastography (MRE) for liver steatosis diagnosis or staging, showed a good accuracy of 0.95, 0.88 and 0.89, respectively, with a MRI specificity of 90.1, while the use of MRS and MRE of 82.1 and 86.4 respectively (20).

The identification of NASH in patients with NAFLD is not possible through MR; however, to detect advanced fibrosis, they are increasingly reliable (20). The MRS and the proton density fat fraction (MRI-PDFF) have been shown to diagnose fibrosis and steatosis in patients with NAFLD accurately, (21,38) additionally, MRS is the most accepted method and it is considered a reference image method for the quantitative analysis of hepatic steatosis, (42,43) with good applicability in patients with obesity or ascites, however, it is costly and may not always be available (20,28). The MRI-PDFF is a recent technique that quantitatively reports the deposition of liver fat, showing a good correlation with MRS and liver biopsy, (42,43) it has an excellent diagnostic accuracy to quantify hepatic steatosis comparing it with the biopsy (Rs = 0.758, p <0.001). The MRI-PDFF can be used to evaluate changes in the long-term degree of steatosis, (44,45) it also allows quantification of the fat fraction in other tissues (40). On the other hand, MRE is more accurate than TE to diagnose liver fibrosis in patients with NAFLD (38). Although MR-based techniques are accurate and useful in patients with obesity, they are more expensive and not widely available compared to ET (38).

**Invasive methods**

**Hepatic biopsy**

Liver biopsy remains the gold standard to confirm the diagnosis and define the severity of NAFLD, a disease characterized by the accumulation of lipids, mainly in macrovesicular form. The histological manifestations vary from mild steatosis, in only 5% of hepatocytes, to more severe forms with lobular and portal inflammation, hepatocyte ballooning and fibrosis in various distribution patterns until the final stage of cirrhosis. NASH is a combination of three findings: steatosis, hepatocyte ballooning, and lobular inflammation (24,46). The accuracy of liver biopsy to evaluate fibrosis has been questioned, mainly due to sampling errors, and intra- and inter-observer variability, which can lead to an over or underestimation of the hepatic fibrosis stage (19). In addition to technical problems, liver biopsy is a costly and invasive procedure that requires surgeons and pathologists to be trained to obtain adequate and representative results, which limits its use for mass screening (28).

The NASH clinical research network has used the following diagnostic categories for NAFLD: without NAFLD (<5% steatosis); NAFLD without NASH (> 5% of steatosis with or without lobular and portal inflammation); NASH borderline zone 3 or NASH borderline zone 1 (most, but not all, the steatohepatitis criteria, with marked steatosis, or lesion in zone 3 or zone 1, respectively) and definitive NASH, all requirements are present, including steatosis, ballooning, and lobular inflammation. Any of these categories may or may not have fibrosis. Specifically, stage 1, is fibrosis in zone 3, perisinusoidal or periportal; stage 2 is indicated if there is periportal fibrosis in zone 3; stage 3 which is indicated, when there is fibrosis from one portal area to another, called bridging fibrosis with nodularity; and finally, stage 4 or cirrhosis (1).

There are two semiquantitative systems for the evaluation of necroinflammatory activity in NAFLD: the NAFLD Activity Score (NAS) of the NASH clinical research network, and the Steatosis Activity Fibrosis (SAF) of the Fatty Liver Inhibition of Progression (FLIP) Consortium (1). NAS is a scoring system that includes three histological features, steatosis, ballooning, and lobular inflammation. Fibrosis is not included as a NAS component (47). The SAF score is designed to diagnose NASH and limit interobserver variation reliably; the algorithm has already been validated for the evaluation of liver damage in patients with morbid obesity subjected to bariatric surgery. The different components of the SAF score (S = steatosis, A = activity, F = fibrosis) have a concordance for steatosis of k = 0.61, k = 0.75 and k = 0.53 respectively. The agreement for the two active components (ballooning and lobular inflammation) is 0.8 and 0.72, respectively (46). There is an excellent agreement between both systems of histological evaluation to diagnose the presence or absence of NASH (47).

A liver biopsy should be considered in patients with NAFLD that were identified as having a high risk of developing steatohepatitis or advanced fibrosis, in addition to presenting concomitant metabolic syndrome or coexisting chronic liver diseases (1).

## Targeted therapy for NAFLD

**Lifestyle modification**

Lifestyle modification consisting of diet, exercise, and weight loss has been recommended to treat patients with NAFLD; in fact, total weight loss is the key to improve NASH histopathologic features (1). A weight loss of at least 3 to 5% improves liver steatosis, while a loss greater of 7% improves the NAFLD activity score; actually, weight loss substantially improves fasting plasma glucose, glucose, and lipid tolerance and could even improve adiponectin levels. The durability of the benefits achieved and the safety of long-term weight loss are still unknown (48). A weight-loss greater than 10% is associated with an improvement in the majority of NASH histopathologic features, including portal inflammation and fibrosis. However, it is crucial to keep in mind that 94% of patients who lost more than 5% of body weight stabilized or improved fibrosis (48).

Frequently, both exercise and diet counseling is recommended for patients with NAFLD to achieve weight loss goals. A combination of a hypocaloric diet (daily reduction by 500-1000 kcal) and moderate-intensity exercise is likely to provide the best likelihood of sustaining weight loss over time (1). The optimal nutritional composition for NAFLD is unknown; however, low carb versus low-fat caloric restriction produced a similar reduction in liver fat and ALT levels (48).

Patients who maintain physical activity more than 150 minutes per week or increase their activity level by more than 60 minutes per week have a more pronounced decrease in serum aminotransferases, regardless of weight loss. The effects of exercise underlying NASH are less clear; but based on a large retrospective assessment of biopsy-proven NAFLD patients, moderate-intensity exercise was not associated with an improvement in the severity of NASH or fibrosis (49).

**Pharmacological strategies**

**Insulin sensitizers**

**Pioglitazone**

Among the drugs that make up the thiazolidinediones group, the most studied for NAFLD treatment is pioglitazone, which improves steatosis and NASH severity, as well as a slight improvement in fibrosis; therefore, it could be used in the management of this entity, but not before warning the patient about the risks and benefits. So far, this option should not be used in NAFLD treatment without the presence of NASH being demonstrated by biopsy (1,49).

**Metformin**

Although several studies have shown an improvement in aminotransferase levels and insulin resistance, metformin fails to improve NAFLD by histology significantly. As well, the American Association for the Study of Liver Diseases (AASLD) practice guidelines do not recommend metformin to treat NASH in adult patients (1).

**Liraglutide**

In a murine model of NAFLD induced by a high-fat diet, liraglutide (a glucagon-like peptide-1 agonist), contributes to appetite suppression and a marked decrease in fat mass. Sirtuins, a family of proteins that play a vital role in autophagy, are probably part of the mechanism that allows liraglutide to improve NAFLD since liraglutide probably acts through the SIRT1/SIRT3-FOXO3a pathway to enhance autophagy (50). Besides, it is suggested that liraglutide enhances the severity of NASH, as well as a slight improvement in the progression to fibrosis, by attenuating oxidative stress and promoting antioxidant processes (49). However, it would be very hasty to consider liraglutide as a strategy for the specific treatment of patients with NAFLD or NASH (1).

**Others**

Oxidative stress is considered to be a mechanism of hepatocellular injury that results in the progression of NAFLD to NASH and fibrosis. Since vitamin E is an antioxidant, its use for NASH treatment has been investigated, (1) where its administration is associated with an improvement of hepatic steatosis in non-diabetic patients (49). According to the clinical practice guideline of the AASLD, the use of vitamin E in non-diabetic patients with NASH demonstrated by biopsy could be considered, after an individualized assessment of the risks and benefits. However, it should be noted that it is not recommended as a treatment strategy in patients with diabetes, with NAFLD without liver biopsy or cryptogenic cirrhosis (1).

On the other hand, statins focus on the management of comorbidities such as diabetes and obesity; however, this is not currently recommended in patients with NASH (49). Other therapies, as ursodeoxycholic acid, are not recommended for the treatment of NAFLD or NASH. Similarly, omega-3 fatty acids should not be used as a specific treatment of NAFLD or NASH; though, it may be considered to treat hypertriglyceridemia in patients with NAFLD (1).

In support of the guidelines published by AASLD, therapies such as metformin, liraglutide, statins, pentoxifylline, and ursodeoxycholic acid are often used off-label; however, due to lack of data, they are currently not recommended as a NASH treatment strategy (49).

**Surgical intervention**

Bariatric surgery is an adequate treatment for obesity management that allows a sustained weight loss and consequently decreases liver steatosis, steatohepatitis, and fibrosis (51,52). Obese people with NAFLD or NASH usually undergo this surgical intervention; however, it is premature to establish bariatric surgery specifically as an option for the treatment of NASH (1).

**Integral assessment of the patient with NAFLD**

**Figure 1:**
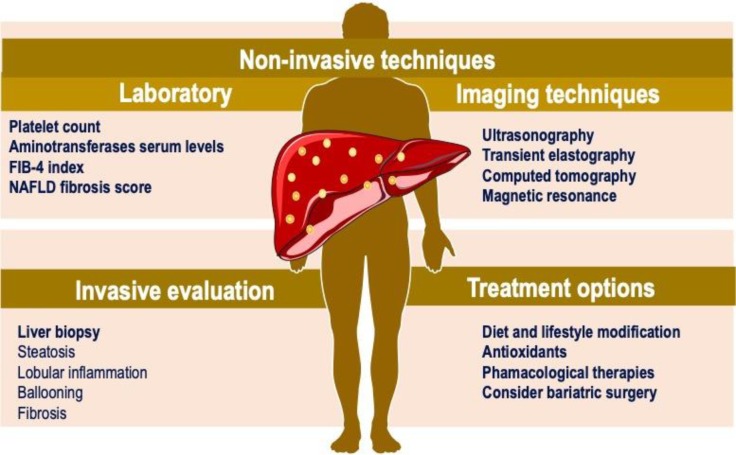
NAFLD diagnosis and treatment approach. Non-invasive techniques, invasive evaluation and treatment options

Determination of a strategy for the diagnostic and treatment approach in the patient with NAFLD is essential in primary care (figure 1). It is important to recognize patients with hepatic steatosis at high risk of developing metabolic and hepatic complications. Therefore, to determine the metabolic state, a biochemical evaluation should be performed, along with the body mass index: (a) lean and metabolically healthy patient, (b) lean but metabolically unhealthy patient, (c) overweight/obese but metabolically healthy patient or (d) overweight/obese and metabolically unhealthy patient (13,53). Follow-up visits to assess liver fibrosis and cardiovascular risk should focus on those patients with metabolic diseases, regardless of their body mass index (53).

All cases in which hepatic steatosis is identified, the patient should be questioned regarding the consumption of steatogenic drugs, alcohol, and traditional medicine to identify possible secondary causes of hepatic steatosis. The high risk of infection with Hepatitis B and C viruses warrants testing for these hepatotropic viruses (1,53).

Alcohol and obesity have an additive effect on cardiovascular risk and liver fibrosis (7). Scores such as the Framingham scale, the algorithm of the American College of Cardiology/American Heart Association and the Globalrisk score should be used in all metabolically ill patients, to determine cardiovascular risk (53,54).

Non-invasive liver fibrosis markers are a fundamental tool in the management of patients with NAFLD (8,10). The rule to select the most useful tools should be their availability and local validation. Considering the previously mentioned techniques seems logical to use two different non-invasive tools for the evaluation of hepatic fibrosis, reserving invasive approaches for cases in which discordant results are obtained. The NFS and TE are the most commonly used techniques for the assessment of liver fibrosis (19,35,53). 

With the aforementioned, the clinician will be able to classify the subject under study in (a) high cardiovascular and hepatic fibrosis risk; (b) high cardiovascular risk and low risk of liver fibrosis; (c) low cardiovascular risk and high risk of hepatic fibrosis; (d) low cardiovascular and liver fibrosis risk. In this way, patients who require further evaluation can be identified, not only by the gastroenterologist but also by the cardiologist or the endocrinologist and initiate a multidisciplinary treatment plan to prevent liver, cardiovascular and metabolic complications (53).

Changes in lifestyle and medical intervention could prevent progression to fibrosis. Thus, timely diagnosis and referral for treatment initiation are of critical importance. Identifying at-risk patients and referring them to initiate treatment could help reduce the long-term consequences of NAFLD; ergo, greater awareness of this association is required in primary care providers, and reproductive health (55).

The management of NAFLD should consist of treating liver disease as well as the associated metabolic comorbidities such as obesity, hyperlipidemia, insulin resistance, and type 2 diabetes mellitus. Pharmacological treatments aimed primarily at improving liver disease should generally be limited to those with biopsy-proven NASH and fibrosis (1). 

## Conflict of interests

Nicolás Salva-Pastor, Norberto C. Chávez-Tapia, Misael Uribe and Natalia Nuño-Lámbarri certify that have no commercial associations (e.g., consultancies, stock ownership, equity interests, patent-licensing arrangements) that might pose a conflict of interest in connection with the submitted article.
